# Neonatal outcomes after the transfer of vitrified blastocysts: closed versus open vitrification system

**DOI:** 10.1186/1477-7827-11-107

**Published:** 2013-11-21

**Authors:** Yuan Chen, Xiaoying Zheng, Jie Yan, Jie Qiao, Ping Liu

**Affiliations:** 1 Department of Obstetrics and Gynecology, Center of Reproductive Medicine, Peking University Third Hospital, Beijing 100191, People’s Republic of China; 2 Key Laboratory of Assisted Reproduction, Ministry of Education, Beijing 100191, China

**Keywords:** Vitrification, Blastocyst, Closed carrier, Open carrier

## Abstract

**Background:**

Increasing evidence indicates that closed vitrification has been successfully used in the cryopreservation of human oocytes and embryos. Little information is available regarding the neonatal outcome of closed blastocysts vitrification. The aim of this study was to evaluate the effectiveness and safety of blastocyst vitrification using a high-security closed vitrification system compared with an open vitrification system.

**Methods:**

A total of 332 vitrified-warmed blastocyst transfer cycles between April 2010 and May 2012 were analyzed retrospectively. The post-thaw survival rate, implantation rate, clinical pregnancy rate, live birth rate, and neonatal outcome were recorded.

**Results:**

There were no significant differences between the open vitrification group and the close vitrification group regarding the post-thaw survival rate (98% versus 95.8%), clinical pregnancy rate (47.6% versus 42.2%), implantation rate (42.9% versus 35.6%), and live birth rate (39.8% versus 32.1%). In total, 332 warming cycles produced 131 healthy babies. There were no significant differences in the mean gestational age, the birth weight, and the birth length between the two groups. No adverse neonatal outcomes were observed in the children born after the transfer of closed vitrified blastocysts compared with the transfer of open vitrified blastocysts.

**Conclusions:**

These data suggest that blastocyst vitrification using a closed vitrification device seems safe and effective with results comparable to those obtained through open vitrification.

## Background

Vitrification has been widely used for oocyte and embryo cryopreservation in assisted reproduction clinics. Better post-thawing survival rates of vitrification compared with the rates of slow-freezing of blastocysts have been demonstrated [1]. Blastocyst vitrification using open carrier, such as a cryoloop, yields comparable clinical outcomes and congenital defect rates as fresh blastocyst transfer [2]. Since an open vitrification system has a theoretically higher risk of microbiological transmission through liquid nitrogen [3-5], there is a movement towards the use of closed vitrification carriers. One concern with a closed vitrification system is whether the lower cooling rate would have an adverse effect on vitrification. The cooling rate with the open carriers has been reported to be superior to -20,000°C/min [6,7], whereas the cooling rate with close carrier is below -2000°C/min [8].

A growing body of evidence indicates that closed vitrification using the CBS High Security straw has been successfully used in the cryopreservation of human blastocysts from the early cavitation stage to the expanded blastocyst stage or derived from biopsied embryos [9,10]. The DNA damage in the blastomeres was comparable in mice embryos vitrified on the open Cryoloop and the closed CBS High Security straw [11]. Little information is available concerning the perinatal outcome of closed blastocysts vitrification. The aim of the present study is to evaluate the clinical efficiency and safety of blastocyst vitrification using a closed device (CBS High Security straws; Cryo Bio System) compared with open device (Cryoleaf).

## Methods

### Study design

The study was approved by the Ethics Committee of the Peking University Third Hospital. A total of 332 blastocysts warming cycles of IVF/ICSI patients performed between April 2010 and May 2012 were analyzed retrospectively. The patients were <40 years old with a BMI (body mass index) <30 kg/m^2^, without previous viral infection (hepatitis B-C, HIV and syphilis). All the patients used long or short protocols for ovarian hyperstimulation. Thirty-six hours after the hCG administration, the oocytes were retrieved and fertilized using conventional IVF or ICSI. Normal fertilization was assessed by the presence of two pronuclei and a second polar body at 16–18 hours after insemination. The zygotes were cultured in cleavage medium (Vitrolife, Sweden) to day 3 and the transfer. The blastocyst vitrification was performed for the patients with the culture of surplus embryos to the blastocyst stage after the fresh day 3 embryo transfer. Only expanded or hatching blastocysts (according to Gardner’s grading system [12]) with ICM (Inner Cell Mass) and trophoetoderm type above grade CC were selected for cryopreservation on day 5 or day 6.

### Vitrification of blastocysts

Prior to the vitrification, the expanded or hatching blastocysts were treated with a laser pulse for the artificial shrinkage of the blastocoelic cavity. The vitrification and warming procedures were similar to those reported by Mukaida T et al. [13]. The solutions were incubated at 37°C for 30 min prior to the vitrification process, and all the steps were conducted at 37°C. The blastocyst was first incubated for 1 min in a droplet of basal medium. The basal medium was Quinns Advantage medium with HEPES (SAGE, Trumbull, CT, USA) supplemented with 20% (v/v) Human Serum Albumin (HSA, Vitrolife, Sweden). The blastocyst was moved to a droplet of equilibration solution composed of 7.5% (v/v) DMSO (Sigma Chemical Co., MO, USA) and 7.5% (v/v) ethylene glycol (Sigma Chemical Co., MO, USA) in basal medium for 2 min. The blastocyst was then transferred into a droplet with vitrification solution containing 15% (v/v) DMSO, 15% (v/v) ethylene glycol and 0.65 mol/L sucrose in a basal medium for 30 s and immediately placed in the McGill Cryoleaf (ORIGIO, Malov, Denmark.) and plunged into liquid nitrogen. As for the closed vitrification, the protocol was similar to that of the open vitrification except for loading the blastocyst onto the closed CBS High Security straws (Cryo BioSystem, Paris, France). The straw was heat sealed and plunged into liquid nitrogen as described previously [9]. The vitrification procedures carried out did not exceed 90 s.

### Warming and recovery of blastocysts

The blastocysts were unloaded from the carrier into warming solution 1 containing 0.33 M of sucrose (Sigma Chemical Co., MO, USA) in a basal medium with 20% HSA. After 2 minutes they were transferred into warming solution 2 containing 0.2 M of sucrose for 3 minutes. Finally, the blastocysts were washed in a droplet of washing solution (HEPES-buffered medium supplemented with 20% HSA) for 5 min. All the warming steps were performed at 37°C. The blastocysts were unloaded from the different carriers as follows: (1) Cryoleaf: the blastocysts were recovered by quickly immersing the sheet in warming solution 1. (2) CBS High Security Straw: the straw was held in liquid nitrogen, and the upper end of the outer straw was cut using wire cutters. The inner straw with a gutter was quickly pulled out of the sheath and immersed in warming solution 1 to unload the embryos. After warming, the blastocysts were transferred to a culture dish with blastocyst medium (G-2, Vitrolife, Sweden) to assess their morphological survival and perform assisted hatching using a laser. The blastocysts with good survival (less than half of the blastocysts showing signs of damage) and the re-expanded blastocysts were transferred 2 h after the in vitro culture. The blastocysts with less than 50% damage but showing no signs of expansion were further cultured for an additional period of 24 h. The transfer will be cancelled if no signs of re-expansion were present.

### Thawed blastocyst transfer

The thawed blastocyst transfer was performed in natural monitored cycles or in programmed artificial cycles. For the natural monitored cycles, the thawed blastocyst transfer was scheduled for 5 days after ovulation. Luteal support was provided with intramuscular injections of progesterone 20–40 mg from the night of transfer. For the hormone replacement therapy, endometrial development was achieved by daily oral estradiol administration. When the endometrial thickness was suitable, this phase was complemented by the administration of progesterone. The blastocyst transfers were performed on day 5 after the initiation of the progesterone treatment. The serum HCG levels were measured 12 days after the transfer.

### Outcome parameters

All of the pregnant women were followed until two months after parturition, and the details of the clinical outcome were obtained from the medical records of our clinic. The blastocyst survival was defined as less than one-half of the blastocysts showing signs of damage. Clinical pregnancy was defined as the presence of gestational sacs observed on an ultrasound scan at least 5 weeks after the embryo transfer. The implantation rate (the number of gestational sacs divided by the number of transferred embryos), the miscarriage rate per clinical pregnancy and the live birth rate per transfer were measured. The neonatal outcomes evaluated were the mean gestational age, gender, preterm birth rate, birth weight, birth length and major and minor anomalies. Low birthweight was defined as birth weight <2500 g. high birthweight was defined as birth weight ≥4000 g. Preterm birth was defined as birth before 37 weeks of gestation. Postterm birth was defined as birth after 42 weeks of gestation.

### Statistics

The data were analyzed by the Chi-square test or Student’s *t*-test. The *P*-values <0.05 were regarded as significant.

## Results

A total of 226 and 106 vitrified-warmed blastocyst transfer cycles were performed for the closed vitrification group and the open vitrification group, respectively. There were no significant differences in maternal age at the time of transfer, BMI, reason for infertility, method of fertilization and average number of transferred embryos between the two groups (P > 0.05, Table 1). Blastocyst survival rate was 98.0% in the open vitrification group and 95.8% in the closed vitrification group. The clinical pregnancy, implantation, miscarriage and live birth rates were similar between the two groups (P > 0.05, Table 2). Three cases of transfers in the closed vitrification group took place 24 hours later, but only one woman became pregnant. The pregnancy complications and spontaneous vaginal delivery rates were comparable between the two groups.

**Table 1:** Clinical parameters of open and closed vitrification

	**Open vitrification**	**Closed vitrification**	**P value**
	**(106 cycles)**	**(226 cycles)**	
*Patient age at transfer (years)*	33.1 ± 4.4	32.5 ± 4.1	0.171
*BMI (kg/m*^*2*^*)*	23.38 ± 3.3	24.19 ± 4.6	0.105
*No. of primary infertility*	68 (64.2)	139 (61.5)	0.643
***Reason for infertility***			
*Female factor*	59 (55.7)	114 (50.4)	0.375
*Male factor*	18 (17.0)	46 (20.4)	0.468
*Others*	29 (27.4)	66 (29.2)	0.729
***Method of fertilization***			
*IVF*	64/106 (60.4)	136/226 (60.2)	0.972
*ICSI*	40/106 (37.7)	86/226 (38.1)	0.956
*Half ICSI*	2/106 (1.9)	4/226 (1.7)	0.941
*Mean number of embryos transferred*	1.39 ± 0.52	1.31 ± 0.53	0.193

Values are mean ± SD or n/total (%).

**Table 2:** Clinical outcomes of open and closed vitrification

	**Open vitrification**	**Closed vitrification**	**P value**
	**(106 cycles)**	**(226 cycles)**	
*Cryosurvival rate*	147/150 (98.0)	295/308 (95.8)	0.224
*Cancellation rate*	3/106 (2.8)	8/226 (3.5)	0.736
*Clinical pregnancy rate/transfer*	49/103 (47.6)	92/218 (42.2)	0.365
*Implantation rate/transferred blastocysts*	63/147 (42.9)	105/295 (35.6)	0.138
***Pregnancy loss***			
*Miscarriage rate/pregnancy*	8/49 (16.3)	21/92 (22.8)	0.363
*Ectopic rate/pregnancy*	0	1/92 (1.1)	1.000
*Live birth rate/transfer*	41/103 (39.8)	70/218 (32.1)	0.176
***Complications during pregnancies***			
*Gestational diabetes*	1/103 (0.97)	3/218 (1.3)	0.760
*Hypertension*	1/103 (0.97)	2/218 (0.92)	0.963
*Placenta previa*	0	1/218 (0.4)	1.000
***Mode of delivery***			
*Spontaneous vaginal delivery*	5/41 (12.2)	10/70 (14.3)	0.756
*Cesarean section*	36/41 (87.8)	60/70 (85.7)	0.756

Values are n or n/total (%).

A total of 132 babies (70 males and 62 females) were born from 111 deliveries as the result of vitrified-warmed blastocyst transfers. There were no significant differences in gender rate and multiple-birth rate (P > 0.05, Table 3). Among the 90 delivered singletons, 30 were derived from the open vitrification group and 60 from the closed vitrification group. Of the 21 women who gave birth to twins after the transfer of two vitrified-warmed blastocysts, 11 were from the open vitrification group and 10 were from the closed vitrification group. For the singleton and twin groups, there were no significant differences in the mean gestational age, the mean birthweight, the mean birth length, the low birthweight rate, and the preterm birth rate between the two vitrification groups (P > 0.05, Table 4). Four babies (three following twin pregnancies) of low birthweight were from the open vitrification group, whereas nine babies (four following twin pregnancies) of low birthweight were from the closed vitrification group. No significant differences in the rate of post-term birth and high birthweight between singletons in the closed vitrification group and singletons in the open vitrification group. A total of 332 warming cycles produced 131 healthy babies. No birth defects occurred among the newborns, except one baby of twins died from fetal distress in the open vitrification group.

**Table 3:** Neonatal parameters of open and closed vitrification

	**Open vitrification**	**Closed vitrification**	**P value**
*Babies born/transferred blastocysts*	52/147 (35.4)	80/295 (27.1)	0.074
*Male rate*	29/52 (55.8)	41/80 (51.3)	0.611
*Female rate*	23/52 (44.2)	39/80 (48.7)	0.611
*Multiple birth rate*	11/41 (26.8)	10/70 (14.3)	0.103
*Singletons*	30	60	
*Twins*	11	10	
*Stillbirth rate /transfer*	1/103 (0.97)	0	0.321

Values are n or n/total (%).

**Table 4:** Neonatal outcome of open and closed vitrification for singletons and twins respectively

	**Singletons**			**Twins**		
	**Open vitrification (n = 30)**	**Closed vitrification (n = 60)**	**P value**	**Open vitrification (n = 11)**	**Closed vitrification (n = 10)**	**P value**
*Gestational age (wks)*	38.3 ± 1.3	37.9 ± 1.5	0.175	37.2 ± 1.4	37.4 ± 1.5	0.844
*GA < 37 weeks*	2/30 (6.7)	9/60 (15.0)	0.426	4/11 (36.4)	4/10 (40)	1.000
*GA ≥ 42 weeks*	1/30 (3.3)	2/60 (3.3)	1.000	0	0	
*Birth weight (g)*	3248 ± 408	3163 ± 481	0.410	2800 ± 380	2598 ± 364	0.091
*<2500 g*	1/30 (3.3)	5/60 (8.3)	0.654	3/11 (27.3)	4/10 (40)	0.659
*≥4000 g*	3/30 (10.0)	7/60 (11.7)	0.813	0	0	
*Birth length (cm)*	49.7 ± 1.6	50.4 ± 1.3	0.086	48.8 ± 1.6	48.9 ± 1.7	0.858

Values are mean ± SD or n/total (%).

## Discussion

These findings demonstrate that a closed human blastocyst vitrification system with artificial shrinkage could be an effective and safe procedure for vitrification.

There have been concerns with the closed system because the slower cooling rate might cause ice crystal formation that could potentially be detrimental to survival. In this study, the closed vitrification protocol, such as the concentration of the cryoprotectants, the temperatures and the exposure times were identical to those described with the open vitrification device. This finding demonstrates that, although a lower cooling rate is a consequence of using a closed vitrification device, it did not affect the vitrification outcome negatively, which might be because of the dominance of the warming rate over the cooling rate in the process of vitrification [14]. In agreement with our data, a recent prospective randomized study that included 432 warming cycles demonstrated that closed or open vitrification with the VitriSafe device rendered comparable clinical results [15]. Although there are no significant differences, most likely because of the small sample size, the indexes of the clinical outcome of open vitrification were most likely better than those of closed vitrification. In this regard, further study is warranted.

Another concern with vitrification is that the risk of detrimental intracellular ice formation is higher in the expanded blastocysts compared with the early blastocysts because of the large fluid-filled cavity. Artificial shrinkage or collapse of the expanded blastocysts by mechanical methods or laser prior to vitrification, as suggested in our study, has been shown to improve survival and increase the clinical pregnancy rates [16]. One published group indicates that the survival rate of full and expanded blastocysts in a closed vitrification system without artificial shrinkage, which was replaced by extended exposure time to the cryoprotectants, is acceptable [9].

Although pregnancies after the transfer of thawed frozen embryos appear to have better obstetric and perinatal outcomes than those after fresh embryos [17,18], the perinatal outcome of vitrified cleavage embryos using the Cryoleaf carrier system yields comparable outcomes with those of the fresh cycles. No significant differences were observed in the mean gestational age, birth weight, sex ratio, congenital birth defects, and abnormalities [19]. The health of children born after blastocyst vitrification has always been of concern. Most studies of blastocyst vitrification are small cases without data on the neonatal outcomes. Wikland M. et al. demonstrated that children born after the transfer of open vitrified blastocysts (using the cryoloop), compared with fresh blastocysts, yields similar neonatal outcomes [20]. The largest study on vitrified blastocysts that included 147 children showed no differences in the obstetric outcomes for children born after open vitrified blastocysts (using the cryoloop) compared with the children born after fresh blastocysts; however, a low birthweight rate of 43.5% among all the children in the vitrified group was reported [2]. There are very few studies on the follow-up of children after transfer of closed vitrified blastocysts. Wirleitner et al. showed that the length of the storage time of vitrified blastocysts in closed devices that were stored for 6 years in liquid nitrogen had no detected negative effect on the health of the children [21]. In this study, 332 warming cycles produced 131 healthy babies. No adverse outcome was observed in the children born after the transfer of closed vitrified blastocysts compared with the children born after the transfer of open vitrified blastocysts in terms of birth weight, birth length or birth defects. In this regard, it is suggested that closed vitrification has an advantage for the cryopreservation of blastocysts derived from patients with viral infections, such as hepatitis B-C and HIV.

## Conclusions

One limitation of this study is its limited size; however, the results are promising. The data suggest that closed blastocyst vitrification seems a safe alternative for open blastocyst vitrification without affecting the efficiency of cryopreservation. Long-term child follow-up studies are needed to investigate open and closed vitrification systems.

### Consent

Written informed consent was obtained from the patient for the publication of this report and any accompanying images.

## Abbreviations

BMI: Body mass index; DMSO: Dimethyl sulfoxide; HCG: Human chorionic gonadotropin; HSA: Human serum albumin; ICM: Inner cell mass.

## Competing interests

The authors declare that they have no conflict of interest.

## Authors’ contributions

All authors participated in the design, interpretation of the studies, analysis of the data and drafted of the manuscript; QJ and LP participated in the design of the study. CY, ZXY, and YJ conducted the experiments; CY wrote the manuscript. LP revised the article critically for important intellectual content. All authors read and approved the final manuscript.
